# A Comparative Evaluation of the Genetic Variant Spectrum in the *USH2A* Gene in Russian Patients with Isolated and Syndromic Forms of Retinitis Pigmentosa

**DOI:** 10.3390/ijms252212169

**Published:** 2024-11-13

**Authors:** Natalya Ogorodova, Anna Stepanova, Vitaly Kadyshev, Svetlana Kuznetsova, Olga Ismagilova, Alena Chukhrova, Aleksandr Polyakov, Sergey Kutsev, Olga Shchagina

**Affiliations:** Research Centre for Medical Genetics, Moscow 115522, Russia; ogorodova@med-gen.ru (N.O.);

**Keywords:** retinitis pigmentosa, Usher syndrome, *USH2A*, inherited retinal diseases, IRD, p.(Trp3955*), p.(Glu4445_Ser4449delinsAspLeu), c.8682-9A>G

## Abstract

Pathogenic variants in the *USH2A* gene are the primary cause of both non-syndromic autosomal recessive inherited retinitis pigmentosa (RP) and the syndromic form, characterized by retinal degeneration and sensorineural hearing loss. This study presents a comparative assessment of the genetic variant spectrum in the *USH2A* gene among Russian patients in two clinical groups. A retrospective analysis was conducted on massive parallel panel sequencing data from 2415 blood samples of unrelated patients suspected of having hereditary retinal diseases. The copy number of *USH2A* exons was determined using the quantitative MLPA method with the MRC-Holland SALSA MLPA kit. Biallelic pathogenic and likely pathogenic variants in the *USH2A* gene were identified in 69 patients (8.7%). In the group of patients with isolated hereditary RP (55 patients), the most frequent pathogenic variants were p.(Glu4445_Ser4449delinsAspLeu) (20.9%), p.(Trp3955*) (15.5%), and p.(Cys934Trp) (5.5%). In patients with the syndromic form (14 patients), the most frequent variants were p.(Trp3955*) (35.7%) and c.8682-9A>G (17.9%). It was found that patients with isolated vision impairment rarely had two “null” variants (17.8%), whereas this was common among patients with both hearing and vision impairment (71.4%) (*p* ≤ 0.05), explaining the severity of the disease and the earlier onset of clinical symptoms in the syndromic form of RP. Ten previously undescribed loss-of-function variants were identified. The estimated prevalence of *USH2A*-associated retinal dystrophy in Russia was 1.9 per 100,000 individuals. The obtained data on the differences in the spectra of genetic variants in the *USH2A* gene in the two studied groups highlight the importance of establishing genotype–phenotype correlations and predicting disease severity, aiming at potential early cochlear implantation and selection of target therapy.

## 1. Introduction

Variants in the *USH2A* gene have been described in patients with autosomal recessive retinitis pigmentosa (RP) (Retinitis pigmentosa 39, OMIM:613809) and Usher syndrome type 2 (Usher syndrome, type 2A, OMIM:276901). RP is the most common inherited retinal disease (IRD) worldwide, with an average prevalence of 1 in 4000 individuals [1,2]. It is characterized by the primary degeneration of photoreceptor cells—rods, followed by the degeneration of cones. The main clinical signs of RP include progressive night blindness (nyctalopia) in childhood or adolescence, visual field constriction, and, eventually, loss of central vision [3]. The age of onset, severity, and clinical course of RP vary significantly [4]. Mutations in the *USH2A* gene account for 8–22% of non-syndromic RP cases [5,6,7].

Usher syndrome, with a prevalence of 3.2 to 6.2 cases per 100,000 individuals, is the most common syndromic form of retinitis pigmentosa [8]. Three clinical forms of Usher syndrome are distinguished. Usher syndrome type 1 is the most severe form, manifesting in congenital sensorineural hearing loss, vestibular dysfunction, and the prepubertal onset of progressive RP. Type 2 is characterized by moderate-to-severe hearing loss, absence of vestibular dysfunction, and a later onset of RP. Usher syndrome type 3 includes a wide variety of vestibular and visual impairments, as well as progressive hearing loss [9]. Usher syndrome type 2 is the most common form (accounting for about two-thirds of patients), and up to 85% of cases are caused by pathogenic variants in the *USH2A* gene (OMIM:*608400) [10,11].

The *USH2A* gene is mapped on chromosome 1q41 and contains 72 exons (NM_206933.4), encoding two alternatively spliced protein isoforms: a short extracellular isoform A (comprising 21 exons and consisting of 1546 amino acids) and a full-length transmembrane isoform B (with 51 additional exons), comprising 5202 amino acid residues [12]. The long isoform of the usherin protein is predominantly expressed in the light-sensitive layer of the retina, specifically in the periciliary membrane of the apical part of the inner photoreceptor segments. The primary role of the protein is in intracellular transport and photoreceptor cell survival [13]. In the inner ear, usherin is localized in the basilar membrane of cochlear hair cells, where it is responsible for the formation and maintenance of stereocilia [14,15]. A large number of mutations in the *USH2A* gene are registered in exons encoding the long isoform of the protein.

The mutation spectrum of the *USH2A* gene is highly heterogeneous and includes over 1700 variants classified as pathogenic or likely pathogenic (LOVD and HGMD databases, version 2024.3, accessed on 5 October 2024). Various types of mutations have been described for the *USH2A* gene, including missense and nonsense variants, small deletions/duplications, splice site mutations, gross deletions/duplications, complex rearrangements, and mutations in the non-coding intronic regions of the gene.

The literature reports differences in the spectrum of genetic variants in the *USH2A* gene between syndromic and isolated IRD forms [16,17,18]. In the non-syndromic form of IRD, “disease-specific” pathogenic variants in the *USH2A* gene have been described (i.e., alleles associated with retinal degeneration without childhood-onset hearing loss) [19]. It is also known that the presence of two loss-of-function (LoF) variants in the *USH2A* gene is primarily associated with the syndromic form of IRD, while other combinations may lead to either isolated RP or the syndromic form [18,20].

The spectrum of genetic variants in the *USH2A* gene varies widely across different ethnic groups. For example, the most common variant in the *USH2A* gene c.2299del (p.(Glu767Serfs*21)) occurs more frequently in patients with presumed Usher syndrome in Danish (45% of mutant alleles), Spanish (15%), and British (31%) patients [17,21,22,23,24]. The pathogenic *USH2A* variant c.8559-2A>G is most common in Chinese and Japanese cohorts (26%) [25,26]. In cases of isolated retinitis pigmentosa in the European patient cohort, the frequent pathogenic variant in the *USH2A* gene was c.2276G>T (p.(Cys759Phe)), with an allele frequency ranging from 6% to 12%, whereas in Chinese patients, the variant c.2802T>G (p.(Cys934Trp)) was found in 11.2% of mutant chromosomes [16].

However, despite the currently available data on the spectrum of mutations in the *USH2A* gene in Russian patients with isolated hearing loss and Usher syndrome [8,27], studies to determine the spectrum of pathogenic and likely pathogenic variants in this gene among patients with hereditary retinal diseases have not been performed. Due to the genetic heterogeneity of this group of hereditary diseases, methods based on massively parallel sequencing are used.

The aim of this study was to analyze the spectrum of genetic variants in the *USH2A* gene among Russian patients with isolated and syndromic forms of inherited retinal diseases and to calculate the overall prevalence of these disease forms caused by mutations in this gene in the Russian Federation, based on both the obtained results and population data.

## 2. Results

Massive parallel panel sequencing data from 2415 unrelated patients with supposed hereditary retinal pathology were analyzed. Pathogenic and likely pathogenic variants in zygosity necessary for disease development were identified in 1107 patients (45.8%). Among them, autosomal recessive forms of inherited retinal diseases (IRD) were found in 792 individuals (71.6%), autosomal dominant retinal degeneration—in 173 individuals (15.6%), and X-linked IRD—in 142 probands (12.8%). Among the autosomal recessive forms, the highest number of confirmed cases involved the *ABCA4* gene (306 patients, 38.6%), *CNGB3* (75 patients, 9.5%), and *USH2A* (69 patients, 8.7%).

**Figure 1:**
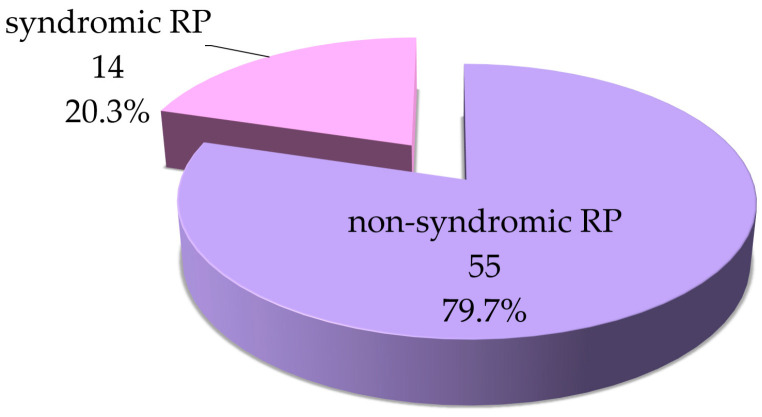
Phenotype distribution among *USH2A*-associated IRD forms.

The high prevalence of *USH2A*-associated retinitis pigmentosa makes a significant contribution to the structure of all autosomal recessive forms of hereditary retinal diseases in Russia. Among the 69 patients with biallelic pathogenic and likely pathogenic variants in the *USH2A* gene, 55 had only visual complaints at the time of the study, while the remaining 14 had both visual impairment and varying degrees of hearing loss, which suggests a possible syndromic form of IRD—Usher syndrome type 2 (Figure 1). The median age of the patients at the time of consultation was 42 years in the group with isolated RP and 27.5 years in the group with both hearing and vision impairment (*p* ≤ 0.05) (Table 1). This age difference is explained by the fact that in the case of Usher syndrome type 2, hearing loss and visual impairment manifest in early childhood, whereas the first complaints of night blindness in patients with isolated RP typically arise in their third decade of life, delaying consultation and diagnostics [20,28,29]. In the current study, the age of clinical symptom manifestation significantly differed between the two groups (*p* ≤ 0.01).

**Figure 2:**
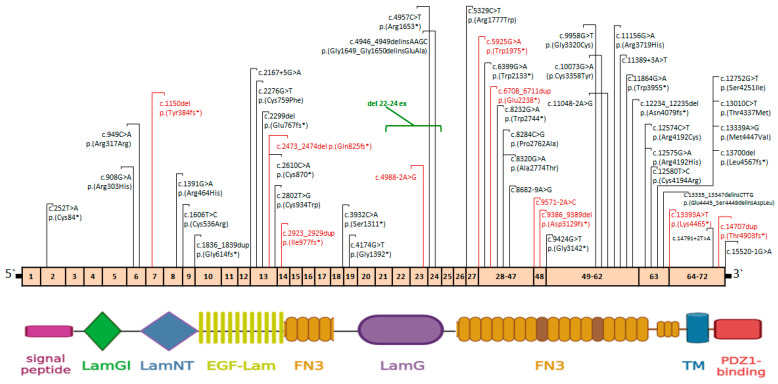
A schematic distribution of pathogenic and likely pathogenic *USH2A* variants was identified in 69 Russian patients with IRD. Previously unreported variants are highlighted in red. The domains of the usherin protein are shown at the bottom. LamGl—laminin G-like domain; LamNT—N-terminal laminin domain; EGF-Lam—epidermal growth factor-like laminin domain; FN3—fibronectin type III domain; LamG—laminin G domain; TM—transmembrane domain; PDZ1-binding—PDZ1-binding domain.

A total of 50 pathogenic and likely pathogenic variants were identified in the *USH2A* gene, 10 of which were previously unreported and represented LoF variants. The distribution of the identified variants in the *USH2A* gene, along with the domain structure of the usherin protein, is presented in Figure 2. The majority of variants are localized in exons 13 and 63, which encode two functionally significant protein domains: EGF-Lam and fibronectin 3, respectively.

**Figure 3:**
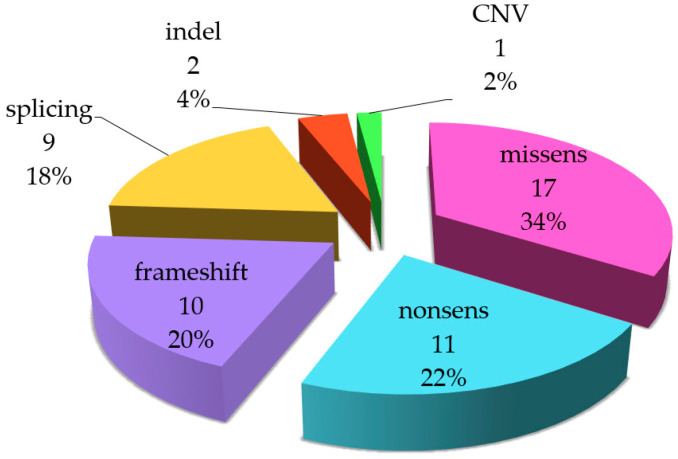
Distribution of nucleotide sequence variants by mutation type in the *USH2A* gene.

Overall, the spectrum of identified nucleotide sequence variants includes 17 missense variants, 12 nonsense, 9 frameshifts, 9 splice site variants, 2 indels, and 1 gross deletion (CNV-copy number variation) (Figure 3).

**Figure 4:**
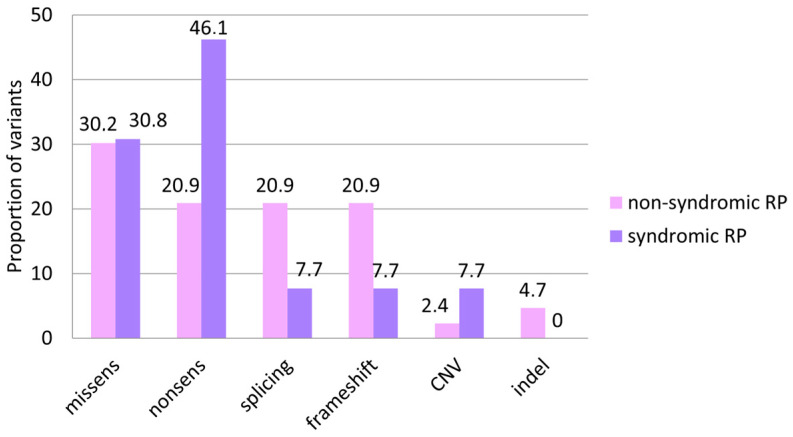
A comparative evaluation of *USH2A* variants by mutation type in two examined groups.

It is known that patients with isolated RP predominantly have missense variants in the *USH2A* gene, whereas patients with the syndromic form often have LoF variants [16,17]. In this study, nonsense variants were found on 20.5% of alleles in patients with non-syndromic RP, whereas in the syndromic form, this proportion increases to 46.2%, though the difference is not statistically significant (*p*-value 0.072). The distribution of alleles by mutation type in the two studied groups is presented in Figure 4.

### 2.1. Spectrum of DNA Sequence Variants in Non-Syndromic USH2A-Associated Retinopathy

Analysis of the spectrum of identified variants in the *USH2A* gene in 55 patients with the isolated RP form showed that the most common pathogenic variants were the previously described c.13335_13347delinsCTTG (p.(Glu4445_Ser4449delinsAspLeu)) and c.11864G>A (p.(Trp3955*)), identified in 23 and 17 chromosomes with mutations, respectively (variant frequencies of 20.9% and 15.5%) (Table 2). These variants were detected in a homozygous state in three patients and one patient, respectively, and both variants in a compound heterozygous state were observed in six probands.

The next most frequent variant, identified on six chromosomes with mutations, was the previously described pathogenic missense variant c.2802T>G (p.(Cys934Trp)) (5.5%), which was found in a homozygous state in one proband. This variant was not observed on chromosomes in the second examined group; however, it has been repeatedly reported in the literature in individuals with both non-syndromic and syndromic retinopathy [30,31].

Other frequent variants in the *USH2A* gene among patients with isolated RP included the previously described pathogenic missense variants c.12574C>T (p.(Arg4192Cys)), c.2276G>T (p.(Cys759Phe)), and the c.4957C>T variant leading to a stop codon, p.(Arg1653*) (each with a frequency of 3.6%). None of these three variants were identified in the second studied group. The first two variants were detected in a compound heterozygous state with the p.(Trp3955*) variant in two and one individuals, respectively. The p.(Cys759Phe) variant was also found once in a homozygous state and was not detected in patients with hearing impairment. The nonsense variant p.(Arg1653*) was observed in two probands in a compound heterozygous state with the p.(Glu4445_Ser4449delinsAspLeu) variant.

Missense variants c.10073G>A (p.(Cys3358Tyr)), c.11156G>A (p.(Arg3719His)), and c.12575G>A (p.(Arg4192His)) have been repeatedly reported in literature in patients with isolated RP [32,33], and were also found in this study in patients with non-syndromic RP (each with a frequency of 2.7%). For example, the c.10073G>A variant was identified in a heterozygous state with the LoF-variants c.11864G>A (p.(Trp3955*)) and c.12234_12235del (p.(Asn4079Trpfs*19)) and in one patient with the c.8284C>G and c.9958G>T variants, which likely represent a complex allele, seeing as they have been reported in a homozygous state in a patient with RP from a Chinese family [34]. These variants (presumably located on the same allele) were also frequently observed in a compound heterozygous state with other pathogenic variants in this gene in patients from China [16].

Additionally, other recurrent LoF variants previously described in the *USH2A* gene were identified in patients with isolated RP: c.13393A>T (p.(Lys4465*)) (2.7%), c.8682-9A>G (2.7%), c.4174G>T (p.(Gly1392*)) (1.8%), and the frameshift variant c.12234_12235del (p.(Asn4079Trpfs*19)) (1.8%).

Previously unreported variants were also identified, most of which are small deletions or duplications leading to a frameshift: c.1150del (p.(Tyr384Ilefs14)), c.14707dup (p.(Thr4903Asnfs51)), c.2473_2474del (p.(Gln825Valfs3)), c.2923_2929dup (p.(Ile977Serfs9)), and c.9386_9389del (p.(Asp3129Glyfs30)). In three patients, a previously unreported nonsense variant c.13393A>T (p.(Lys4465)) was found in a heterozygous form, and the c.5925G>A (p.(Trp1975*)) variant was identified in a single case. The identified nonsense variants, as well as the small deletions/duplications leading to a frameshift, result in a premature termination codon and subsequently trigger nonsense-mediated mRNA decay, which is a known pathogenic mechanism for the *USH2A* gene [35]. Variants affecting the canonical splice acceptor site were found on two chromosomes: c.4988-2A>G and c.9571-2A>C; both variants were classified as likely pathogenic.

Furthermore, among all patients with supposed RP who were referred for testing, 11 were found to have two variants in the *USH2A* gene, one classified as pathogenic/likely pathogenic and the other as a variant of uncertain clinical significance (VUS), seeing as segregation analysis could not be conducted within families (Table 3). If these variants are confirmed to be in a trans configuration, they could be reclassified as likely pathogenic, according to ACMG guidelines [36]. These variants affect amino acid residues in functional protein domains, where other missense variants have previously been described as pathogenic. For two other variants, c.4627+5G>A and c.7594+6T>C, located near the canonical splice site, functional analysis is necessary to establish their clinical significance, as neither variant is reported in control cohorts (GnomAD and ExAC), and prediction programs spliceAI (scores of 0.83 and 0.63, respectively), NetGene2, and Human Splicing Finder 3.0 predicted a pathogenic effect on splicing. At the time of evaluation, these patients did not exhibit hearing impairment, although they had clear signs of retinitis pigmentosa (nyctalopia, visual field constriction, reduced vision acuity, and pigment deposits in the form of “bone spicules” detected during fundus examination).

### 2.2. Spectrum of USH2A Gene Variants Identified in Patients with Syndromic RP

In the group of patients with simultaneous vision and hearing impairment (14 individuals), the most frequent pathogenic variants in the *USH2A* gene were the previously described LoF-variants c.11864G>A (p.(Trp3955*)) (variant frequency 35.7%), c.8682-9A>G (17.8%), and c.9424G>T (p.(Gly3142*)) (7.1%). The first two variants were found in a compound heterozygous state in three patients. Each of the other variants, including c.1606T>C (p.(Cys536Arg)), c.6708_6711dup (p.(Glu2238*)), c.3932C>A (p.(Ser1311*)), c.10073G>A (p.(Cys3358Tyr)), c.252T>A (p.(Cys84*)), c.5329C>T (p.(Arg1777Trp)), c.908G>A (p.(Arg303His)), c.2299del (p.(Glu767Serfs21)), and c.2610C>A (p.(Cys870)), were identified once in the studied cohort (Table 2). The c.6708_6711dup variant had not been previously described in the literature and was not found in population databases, while the remaining variants have been repeatedly reported in patients with syndromic and non-syndromic RP.

In two patients, a gross heterozygous deletion spanning exons 22–24 was identified. This deletion, in combination with other pathogenic variants, was observed in three patients with isolated RP. Among the patients in this group, 10 had two LoF-variants, while the remaining four had a LoF-variant in a compound heterozygous state with a previously described missense variant.

Thus, comparing the genotypes of patients in the two examined groups, we can note that Russian patients with the non-syndromic form of RP more frequently had LoF-variants in combination with missense substitutions (67.3%). In contrast, patients with syndromic RP had a statistically significant predominance of genotypes with two LoF-variants (71.4%) (*p* < 0.05), and no genotypes with two missense variants were observed, which explains the severity of clinical manifestations in the group of patients with RP and sensorineural hearing loss (Table 4).

Based on the contribution of the two most common *USH2A* gene variants (p.(Glu4445_Ser4449delinsAspLeu) and p.(Trp3955*)) to the structure of hereditary retinal pathology in Russian patients, the disease frequency associated with the *USH2A* gene was calculated to be 1.9 per 100,000 individuals (using Hardy–Weinberg equilibrium). Considering that the number of newborns in Russia in 2023 was 1,100,000, an average of 20 children are born each year with *USH2A*-associated RP. The obtained results are of significant importance during the initial stages of examining patients with vision and hearing impairments.

## 3. Discussion

*USH2A*-associated retinal pathology is the third most common autosomal recessive form of IRD among Russian patients, after Stargardt disease, caused by biallelic variants in the *ABCA4* gene and achromatopsia, associated with mutations in the *CNGB3* gene [37]. Pathogenic variants in the *USH2A* gene account for over half of the syndromic cases of retinitis pigmentosa.

In the retina, the function of the usherin protein is to maintain photoreceptor cells, while in the auditory system, its primary role is to maintain stereocilia. For instance, in a study on zebrafish (*Danio rerio*), researchers demonstrated that the long isoform of the protein is located at the connecting synapse of the photoreceptor cilium, and the knockout of the gene led to photoreceptor degeneration [38,39]. In the cochlea, usherin protein exists in two isoforms during early stereocilia development, and functional studies in mice have shown that gene knockout results in non-progressive sensorineural hearing loss [15]. The prevalence of LoF variants in the *USH2A* gene among patients with syndromic forms of IRD correlates with early onset and severe progression of the disease. The presence of two such variants in a compound heterozygous state leads to the absence of residual usherin protein activity in the hair cells of the cochlea and in photoreceptors.

In the current study, the median age at the time of applying for genetic consultation was higher for patients with isolated visual impairments than that of patients who also had auditory problems, consistent with other literature reports [28].

In Russian patients with isolated RP, common variants in the *USH2A* gene were identified: p.(Glu4445_Ser4449delinsAspLeu) and p.(Trp3955*) (with frequencies of 20.9% and 15.5%, respectively). The p.(Trp3955*) variant has been repeatedly described in patients with retinitis pigmentosa and Usher syndrome type 2 from various countries, as well as in cases of isolated hereditary hearing loss [27,40,41]. In a previously studied cohort of Russian patients with Usher syndrome, the p.(Trp3955*) variant was found in 30% of mutant chromosomes, while in patients with hereditary hearing loss, this variant accounted for half of all mutant alleles of the gene. In this study, among patients with the syndromic form of IRD, the p.(Trp3955*) variant was also highly prevalent, accounting for 35.7% of mutant alleles (with an overall frequency of 19.6% in the *USH2A* gene).

The accumulation of this pathogenic variant in Central Europe is attributed to the founder effect. The proportion of patients carrying the p.(Trp3955*) variant was significantly lower in Germany (20%, χ^2^ = 5.3, *p* = 0.022), France (4.9%, χ^2^ = 31.3, *p* < 0.001), and Italy (11.1%, χ^2^ = 8.9, *p* = 0.003) compared to Russia (39.1%) [42]. However, this variant is especially prevalent in the Slovenian population, where it is found in 82.5% of patients, which is significantly higher than in Russia (χ^2^ = 19.2, *p* < 0.001). The high prevalence of the nonsense variant p.(Trp3955*) in Slovenian patients is likely due to multiple unrelated founders as a result of migration from neighboring populations, seeing as four distinct haplotypes associated with this variant have been identified [43].

The frequent variant p.(Glu4445_Ser4449delinsAspLeu) was not found in the chromosomes of patients with the supposed syndromic form of RP and, according to the literature, has not been described in Usher syndrome type 2. This suggests that it may be “specific” to isolated RP in Russian patients. This variant leads to the in-frame deletion of five amino acids and the insertion of two others, disrupting the conservative region within the fibronectin type III (FN3) domain [13]. The variant was first described by Terri L. McGee et al. in 2010 in a patient with retinitis pigmentosa from the United States [41]. According to the literature, this complex rearrangement has not been reported in any population study with a high allelic frequency comparable with that observed in this research (allele frequency 20.9% among patients with non-syndromic RP).

Other common previously described pathogenic variants in patients with non-syndromic RP included p.(Cys934Trp) (5.5%) and p.(Arg4192Cys), p.(Cys759Phe), and p.(Arg1653*) (each accounting for 3.6%). The p.(Cys934Trp) variant is located in a region encoding the functionally and evolutionarily conservative EGF-Lam domain (laminin-type epidermal growth factor-like domain), which is involved in protein stabilization and folding [19]. This variant has been repeatedly reported in the literature in individuals with both non-syndromic and syndromic forms of IRD [30,31] and is common in patients from East Asia (variant frequency in gnomAD is 0.25%). In the current study, the p.(Cys934Trp) variant was not found in patients with hearing impairment.

It is known that symptoms of isolated RP are more common when missense variants such as p.(Arg4192Cys) or p.(Cys759Phe) are observed in a compound heterozygous state with other pathogenic missense variants [33,44]. Enrichment of the European allele with the p.(Cys759Phe) variant has been noted in the group of patients with isolated retinopathy [45]. This variant has a higher reported prevalence in RP (25.4% of *USH2A* variants) than in syndromic forms RP (2.8%) in a sample of patients from the Netherlands and Belgium [20], which is significantly higher than in Russia (χ^2^ = 21.6, *p* < 0.001). Aller E. et al. identified two haplotypes associated with this variant, the most common among Spanish patients with RP [46]. However, population data indicate that the p.(Cys759Phe) variant is less frequent in Russians compared to Europeans, suggesting ethnic differences in the distribution of common variants across European countries, likely due to evolutionary events such as “genetic drift”.

Other missense variants, including p.(Cys3358Tyr), p.(Arg3719His), and p.(Arg4192His) (each accounting for 2.7%), have also been repeatedly described in patients with retinitis pigmentosa from other countries [32,33]. These variants were not found in patients with both hearing and vision impairments, which may indicate that these variants are specific to isolated retinal pathology in Russian patients as well. In the study by Li W. et al., the p.(Arg3719His) variant was common in the Chinese population among patients with isolated RP (allele frequency 2.7%), with no significant differences observed with our results (χ^2^ = 0.14, *p* > 0.05) [47].

The obtained data indicate a high prevalence of pathogenic missense variants among Russian patients with isolated retinopathy. Among all patients with identified biallelic variants, 37 probands had one LoF-variant and one missense variant, while 9 had two missense variants. However, the literature describes cases of syndromic RP where hearing loss in patients occurred at a later age if missense variants were present on the second allele [19,22]. This does not exclude the possible influence of environmental factors, genetic modifiers, and epigenetics.

It has been repeatedly reported that the complete absence of usherin protein due to two truncated LoF-variants has a more detrimental impact on hearing and vision physiology compared to cases where one or two missense variants resulted in an altered form of usherin. Nine patients with isolated RP were found to have two LoF-variants, requiring further monitoring of these patients and the use of comprehensive diagnostic approaches, including audiological methods aimed at the early detection of hearing impairments.

A comparison of the genotypes of Russian patients in the two studied groups revealed that patients with non-syndromic RP more frequently had LoF-variants combined with missense variants (67.3%) and, in some cases, two missense variants (16.35%). In contrast, patients with syndromic IRD predominantly had two LoF-variants (71.4%) and did not have two pathogenic missense variants. This statistically significant finding highlights the higher proportion of LoF-variants in the group of patients with syndromic RP and corresponds with the pathogenesis and severity of the clinical presentation of the disease [29].

In the examined patient cohorts, five individuals were found to have a gross deletion encompassing exons 22–24 of the *USH2A* gene, which was identified using quantitative MLPA analysis. The exact deletion boundaries were subsequently determined as chr1.216259403_216323159del (hg19) via direct Sanger sequencing. The frequency of this pathogenic allele in the *USH2A* gene was 4.4%. The deletion of exons 22 to 24 had previously been reported in Russian patients with isolated hearing loss, although its exact boundaries had not been defined [27]. Additionally, this pathogenic variant has been recurrently observed in German patients with Usher syndrome type 2 [40].

When comparing the spectrum of variants with other population-based studies, the most common *USH2A* gene variant in European countries, c.2299del (with allele frequency varying from 7% to 45%), was detected in this study in only two patients with isolated and syndromic forms of RP (the variant’s frequency in the *USH2A* gene was 1.4%). The c.2299del variant leads to premature termination of translation; however, Lenassi et al. hypothesized that this variant may affect the splicing of exons 12 and 13 of the *USH2A* gene [48]. The widespread geographical distribution of the c.2299del variant is attributed to an ancestral mutation that originated in Southern Europe [49]. Notably, this pathogenic variant was not observed in Slovenian patients with Usher syndrome type 2 [42]. Given that the c.2299del allele is the most common disease-causing allele in European countries, our findings highlight the differences in the spectrum of pathogenic *USH2A* gene variants between Russia and other countries.

The c.8682-9A>G variant, previously described in patients with Usher syndrome and retinitis pigmentosa from Germany [40] and the United States [50], is common among Russian patients with the syndromic form of RP (variant frequency—17.9%). This variant was also found in a heterozygous state in three patients with isolated RP. It is noteworthy that the c.8682-9A>G variant was not detected in Mexican or Chinese patients with the syndromic form of RP [25,26]. This variant was encountered in 0.9% of mutant alleles of the *USH2A* gene in Dutch and Swedish patients with Usher syndrome type IIa, which is significantly lower than in Russia (χ^2^ = 26, *p* < 0.001) [51]. All of this indicates a high prevalence of this pathogenic allele in Russia compared to other ethnic groups.

The identified variants in the *USH2A* gene were relatively evenly distributed across the entire protein. However, pathogenic variants in Russian patients were more frequently found in exons 63 (18%) and 13 (10%) (Figure 2). In the study by Bing-Nan Su et al., a population-based analysis of the genetic spectrum of the *USH2A* gene was conducted to identify “hot spots” associated with *USH2A*-related retinitis pigmentosa. The mutation frequency detected in exon 13 was the highest (18.4%), followed by exons 63 (10%) and 61 (5.5%). Exon 13 contained the most common variants: c.2299del, c.2276G>T, and c.2802T>G [29]. Most variants of the *USH2A* gene affect amino acid residues located in Laminin EGF-like domains and Fibronectin 3 (FN3) domains. Laminin EGF-like domains are thought to play a role in proper protein folding, while Fibronectin 3 domains are found in extracellular proteins and are involved in the spatial organization of other domains and in mediating protein–protein interactions [52].

Despite the heterogeneous spectrum of identified *USH2A* gene variants in Russian patients, a significant number of cases remain without a confirmed diagnosis. Due to the large size of the *USH2A* gene, a substantial number of missense variants of uncertain clinical significance are detected, making their interpretation challenging without additional functional analysis methods. It is also important to note the limitations of panel sequencing, such as the inability to detect structural rearrangements, gross deletions, and duplications, as well as variants located deep within non-coding regions of the gene. For instance, the intronic variant c.7595-2144A>G, previously described in European patients, which leads to the insertion of 152 bp at the junction of exons 40 and 41 and the formation of a premature stop codon p.(Lys2532Thrfs*56) [53], was not observed in this study. However, this does not exclude the presence of other intronic variants specific to the Russian cohort. Therefore, the use of whole-genome sequencing in the molecular diagnosis of retinitis pigmentosa will enhance the informativeness of genetic studies for hereditary retinal disorders and expand the spectrum of genetic variants, which provides the potential for future target therapy.

## 4. Materials and Methods

To analyze the spectrum of genetic variants in the *USH2A* gene among Russian patients, a retrospective analysis of massively parallel sequencing data from 2415 blood samples was conducted. This study included patients with the following diagnoses: “retinitis pigmentosa”, “Stargardt disease”, “achromatopsia”, “Leber congenital amaurosis”, and “cone/cone–rod dystrophy” from various regions of the country. Among the patients, 1146 were female (47.5%) and 1269 were male (52.5%).

DNA was extracted from peripheral blood leukocytes using the QIAamp DNA Mini Kit (Qiagen, Hilden, Germany) according to the manufacturer’s protocol. All patients underwent genetic testing based on massively parallel sequencing with the “Ophthalmo” panel, which includes 212 genes (Supplementary Materials Table S1). The “Ophthalmo” panel spans 822,786 bp and includes 4423 amplicons ranging in size from 125 to 275 bp. The estimated coverage of coding regions, according to the Ion AmpliSeq Designer program, was 97.73%. Sequencing data processing was carried out using the “NGS-Data” information system (https://ngs-data-ccu.epigenetic.ru, accessed on 5 October 2024) [54]. To assess the population frequencies of identified variants, we used data from the “1000 Genomes” project, ESP6500, The Genome Aggregation Database v2.1.1, and RuExac (data based on 2910 exomes sequencing of Russian patients with various hereditary diseases, collected by the Research Centre for Medical Genetics).

The identified genetic changes were called according to the international HGVS nomenclature (http://www.hgvs.org/mutnomen/, accessed on 5 October 2024). To evaluate the clinical relevance of identified variants, the OMIM database, the HGMD^®^ Professional pathogenic variant database version 2024.3, and literature were used. VarSome [55] and Franklin (https://franklin.genoox.com, accessed on 5 October 2024) in silico tools were used to assess the pathogenicity of variants. The clinical significance of previously undescribed nucleotide sequence variants was assessed based on the international sequencing data interpretation guidelines [36]. A family segregation analysis of the identified *USH2A* variants was not conducted due to the absence of biological material from family members.

For patients with *USH2A*-associated retinopathy, medical records were reviewed to collect information on symptoms and other clinical data, such as age, gender, family history, and the age of onset of night blindness and/or hearing loss (Supplementary Materials Table S3). To date, there is no clear methodological distinction between how patients with RP and Usher syndrome were analyzed. Patients were referred from various medical centers and reviewed by different ophthalmologists. Most patients did not have audiologic testing data, so the division of patients is rather tentative.

For patients with only one identified pathogenic/likely pathogenic variant in the *USH2A* gene, copy number analysis of exons 1–72 (NM_206933.3) of the *USH2A* gene was carried out using the quantitative MLPA (multiplex ligation-dependent probe amplification) method with the MRC-Holland SALSA MLPA probemix P361 USH2A mix 1 and P362 USH2A mix 2 kits (https://www.mrcholland.com/, accessed on 5 October 2024). MLPA was performed according to the manufacturer’s protocol using 50 ng of genomic DNA per reaction, and 1 μL of each reaction product was separated on an ABI 3500 xL capillary sequencer (Applied Biosystems, Waltham, MA, USA). The results were analyzed using Coffalyser.Net™ software (https://www.mrcholland.com/technology/software/coffalyser-net/, accessed on 5 October 2024) (MRC-Holland, Amsterdam, the Netherlands). A probe dosage quotient value of 1 ± 0.4 was considered normal; less than 0.6 was considered a deletion, and more than 1.4 a duplication.

In the same patients, a search for the previously described pathogenic intronic variant c.7595-2144A>G in the *USH2A* gene was conducted using allele-specific MLPA, with subsequent detection of the PCR product in 9% polyacrylamide gel. The sequences of oligonucleotides used in the ligation and PCR reactions are provided in the Supplementary Materials Table S2.

Written informed consent was obtained from legal representatives of all patients under 18 years of age and from patients over 18 years of age for participation in the study. This study was conducted in accordance with the guidelines of the Helsinki Declaration and was approved by the Ethics Committee of the Research Centre for Medical Genetics.

Statistical analysis was performed using the Statistica 10 software (StatSoft, Inc., Tulsa, OK, USA). The statistical processing of the data, after the test for equality of distribution normality, has proved that the groups do not meet the normal distribution rule (the groups differed in the number of patients, and the patients had a wide range of ages); that is why the non-parametric test has been used for comparison. Mann–Whitney U-test was used to compare the age of patients and the age of disease onset in each group studied. Fisher’s exact test was applied to compare genetic variants between the samples. A *p*-value of ≤0.05 was considered statistically significant.

## 5. Conclusions

This study describes the spectrum of genetic variants in the *USH2A* gene that cause syndromic and isolated RP in Russian patients. The application of mass parallel sequencing methods significantly contributes to the study of clinical heterogeneity in inherited retinal diseases, particularly retinitis pigmentosa. The presence or absence of congenital hearing loss is a key clinical feature that affects patient monitoring and quality of life, highlighting the need for a comprehensive diagnostic approach that includes audiological examination methods.

In this study, Russian patients with isolated retinal pathology rarely had two “null” variants, whereas this was common in patients with supposed syndromic forms of retinitis pigmentosa (16.35% versus 71.4%, *p* ≤ 0.05). Severe clinical symptoms in the syndromic form are associated with an earlier onset of auditory and visual impairments compared to isolated retinopathy. Among patients with isolated RP, a frequent “retina-specific” variant, p.(Glu4445_Ser4449delinsAspLeu), was identified in 20.9% of cases, with a significantly higher frequency compared to other countries. In patients with the syndromic form of the disease, two common variants, p.(Trp3955*) and c.8682-9A>G, accounted for 53.6% of mutant alleles in the *USH2A* gene. The c.2299del variant, prevalent in European countries, was detected in only two patients in this study (allele frequency of 1.4%). Ten previously undescribed variants leading to loss of protein function were identified in Russian patients, along with a frequent gross rearrangement—deletion of exons 22–24, with a frequency of 4.4%. The estimated prevalence of *USH2A*-associated RP in Russia was calculated to be 1.9 per 100,000 individuals.

The identification of common pathogenic variants in the *USH2A* gene and the establishment of genotype–phenotype correlations will facilitate the optimization of molecular genetic diagnostics at the early stages of patient examination. The information about the genetic variant spectrum in the *USH2A* gene will improve the efficiency and speed of molecular diagnostics by developing simpler and cheaper detection methods of frequent pathogenic variants in a relatively short time of analysis, not comparable to massively parallel sequencing. The results of genetic testing may also contribute to the development of personalized audiological monitoring, rehabilitation, and therapeutic programs. The obtained information will allow the planning of the development of gene therapy in accordance with the spectrum and types of identified mutations in the *USH2A* gene in Russian patients.

## Figures and Tables

**Table 1:** A characteristic of patient groups with non-syndromic and syndromic RP forms.

Criteria	Non-Syndromic RP (N = 55)	Syndromic RP (N = 14)	*p*-Value **
Age (median, IQR *), years	42 (35–49)	27.5 (16–39.5)	0.015
Number of male/female patients	28/27	9/5	-
Age of vision impairment manifestation (nyctalopia/visual field constriction) (median, IQR), years	27 (16–30)	11 (8–18)	0.0096
Age of hearing impairment manifestation (median, IQR), years	-	5 (3–7)	-

N—number of patients; * IQR—interquartile range; ** Mann–Whitney U-criterion.

**Table 2:** Pathogenic and likely pathogenic variants in the *USH2A* gene (NM_206933) with a frequency higher than 1% in patient cohorts with non-syndromic and syndromic RP.

Exon No	cDNA	Effect	Number of Alleles (%)	Frequency in Gnomad *	Frequency in RuExac ***
Non-syndromic RP					
63	c.13335_13347delins CTTG	p.(Glu4445_Ser4449delins AspLeu)	23 (20.9)	0.00016	0.00017
61	c.11864G>A	p.(Trp3955*)	17 (15.5)	0.00024	**0.0014**
13	c.2802T>G	p.(Cys934Trp)	6 (5.5)	n/d, 0.0025 **	n/d
13	c.2276G>T	p.(Cys759Phe)	4 (3.6)	0.0014	**0.00034**
63	c.12574C>T	p.(Arg4192Cys)	4 (3.6)	0.00007	n/d
24	c.4957C>T	p.(Arg1653*)	4 (3.6)	0.000007	n/d
51	c.10073G>A	p.(Cys3358Tyr)	3 (2.7)	0.00076	0.00034
57	c.11156G>A	p.(Arg3719His)	3 (2.7)	0.000008 0.0002 **	**0.0005**
63	c.12575G>A	p.(Arg4192His)	3 (2.7)	0.00012	0.00017
63	c.13393A>T	p.(Lys4465*)	3 (2.7)	n/d	0.00017
int43	c.8682-9A>G	splicing	3 (2.7)	0.0001	0.00034
22–24	del 22–24 ex	gross deletion	3 (2.7)	n/d	n/d
19	c.4174G>T	p.(Gly1392*)	2 (1.8)	n/d	0.00017
62	c.12234_12235del	p.(Asn4079Trpfs*19)	2 (1.8)	0.000018	0.00017
42/50	c.[8284C>G;9958G>T]	p.[(Pro2762Ala); (Gly3320Cys)]	2 (1.8)	n/d	n/d
**Syndromic RP**					
61	c.11864G>A	p.(Trp3955*)	10 (35.7)	0.00024	**0.0014**
int43	c.8682-9A>G	splicing	5 (17.8)	0.0001	0.00034
48	c.9424G>T	p.(Gly3142*)	2 (7.1)	0.00005	0.00017
22–24	del 22–24 ex	gross deletion	2 (7.1)	n/d	n/d
2	c.252T>A	p.(Cys84*)	1 (3.6)	n/d	n/d
6	c.908G>A	p.(Arg303His)	1 (3.6)	0.00006	0.00017
9	c.1606T>C	p.(Cys536Arg)	1 (3.6)	0.000046	n/d
13	c.2299del	p.(Glu767Serfs*21)	1 (3.6)	0.00093	0.00017
13	c.2610C>A	p.(Cys870*)	1 (3.6)	0.00003	n/d
18	c.3932C>A	p.(Ser1311*)	1 (3.6)	0.000009	n/d
27	c.5329C>T	p.(Arg1777Trp)	1 (3.6)	n/d 0.000032 **	n/d
35	c.6708_6711dup	p.(Glu2238*)	1 (3.6)	n/d	n/d
51	c.10073G>A	p.(Cys3358Tyr)	1 (3.6)	0.00076	0.00034

*—variant frequency in the gnomAD database (v2.1.1) in a European population, **—variant frequency in an Eastern Asian population, ***—variant frequency in the RuExAC database (data based on 2910 exomes of Russian patients with various hereditary diseases), n/d—no data. Statistically significant differences in frequency are highlighted in bold (*p* ≤ 0.05).

**Table 3:** Unresolved cases of *USH2A* variants in patients with RP.

Patient No	Sex	Age	1st Variant (Pathogenic/Likely Pathogenic)			2nd Variant (VUS)		
			cDNA	Effect	Exon	cDNA	Effect	Exon
1	m	34	c.2298T>A	p.(Cys766*)	13	c.2800T>C	p.(Cys934Arg)	13
2	f	49	c.13374del	p.(Glu4458Aspfs*3)	63	c.1679C>T	p.(Pro560Leu)	10
3	m	46	c.6325+1G>A	splicing	32	c.13339A>G	p.(Met4447Val)	63
4	f	49	c.11864G>A	p.(Trp3955*)	61	c.15020C>T	p.(Pro5007Leu)	69
5	f	39	c.2276G>T	p.(Cys759Phe)	13	c.1814G>A	p.(Cys605Tyr)	10
6	f	46	c.12574C>T	p.(Arg4192Cys)	63	c.4946_4949delinsAAGC	p.(Gly1649_Gly 1650delinsGluAla)	24
7	f	54	c.10073G>A	p.(Cys3358Tyr)	51	c.1655G>T	p.(Cys552Phe)	10
8	m	44	c.2299del	p.(Glu767Serfs*21)	13	c.14221C>G	p.(Pro4741Ala)	65
9	f	55	c.13335_13347delinsCTTG	p.(Glu4445_Ser4449delinsAspLeu)	63	c.1391G>T	p.(Arg464Leu)	8
10	f	47	c.13335_13347delinsCTTG	p.(Glu4445_Ser4449delinsAspLeu)	63	c.4627+5G>A	splicing?	int21
11	f	67	c.5046C>A	p.(Tyr1682*)	25	c.7594+6T>C	splicing?	int40

**Table 4:** Distribution by genotype in patient groups with non-syndromic and syndromic RP.

Genotype	Non-Syndromic RP	Syndromic RP	*p*-Value **
LoF */LoF	9 patients (16.35%)	10 patients (71.4%)	0.00015
LoF/missense	37 patients (67.3%)	4 patients (28.6%)	0.014
missense/missense	9 patients (16.35%)	-	-

*—LoF-variants include premature termination codon variants, small deletions/duplications leading to a frameshift, splice site variants, and gross deletions. **—exact F-test.

## Data Availability

Data is contained within the article.

## Supplementary Materials

The following supporting information can be downloaded at: https://www.mdpi.com/article/10.3390/ijms252212169/s1.
